# Multifunctional Hydrogel-Based Scaffolds: Integrating Conductive Nanomaterials for Smart Wound Healing Applications

**DOI:** 10.3390/gels12060501

**Published:** 2026-06-04

**Authors:** Myoung Joon Jeon, Youjin Seol, Youjin Jeong, Sayan Deb Dutta, Ki-Taek Lim

**Affiliations:** 1 Department of Biosystems Engineering, Kangwon National University, Chuncheon-si 24341, Gangwon-do, Republic of Korea; myoungjoon.jeon@gmail.com (M.J.J.); yj.seol1008@gmail.com (Y.S.); ujuj021104@gmail.com (Y.J.); sayan91dutta@gmail.com (S.D.D.); 2 Interdisciplinary Program in Smart Agriculture, Kangwon National University, Chuncheon-si 24341, Gangwon-do, Republic of Korea; 3 Institute of Forest Science, Kangwon National University, Chuncheon-si 24341, Gangwon-do, Republic of Korea

**Keywords:** hydrogels, wound healing, conductive nanomaterials, 3D printing, biosensors, tissue engineering

## Abstract

Effective wound management remains a critical challenge in modern medicine, requiring a delicate balance among infection control, hemostasis, and tissue regeneration. Biopolymer-based hydrogels have emerged as leading candidates for medical use due to their biocompatibility, moisture-retention capabilities, and structural similarity to the natural ECM. This review provides a comprehensive overview of the transition from passive dressings to intelligent, multifunctional hydrogel scaffolds. We first examine the biological mechanisms of wound healing and the fundamental roles of hydrogels in maintaining an optimal microenvironment. Central to this discussion is the integration of conductive materials (including conductive polymers, carbon-based nanomaterials, and metal nanoparticles), which empower hydrogels with bio-sensing and electromechanical stimulation capabilities. Furthermore, we explore how 3D printing technologies enable the fabrication of personalized, high-precision scaffolds. The review also discusses the emerging role of integrated monitoring systems and machine learning algorithms in enhancing diagnostic accuracy. By synthesizing current research, this review identifies critical engineering hurdles and outlines the future trajectory toward automated, closed-loop wound-care systems in clinical practice. Ultimately, while these advanced electronic scaffolds offer revolutionary therapeutic paradigms, this review underscores that balancing electroconductivity with chronic cytocompatibility, refining multi-modal biosensor calibration, and navigating complex regulatory evaluation pathways remain critical prerequisites. Overcoming these fundamental translational bottlenecks is essential to realizing the next generation of automated clinical wound care.

## 1. Introduction

The skin acts as a critical interface that protects the human body from diverse environmental hazards. When this biological barrier is disrupted by trauma or chronic disease, its protective capacity is severely diminished. In extreme cases, extensive skin loss can lead to systemic failure or even mortality [1]. Effective wound management must address two primary challenges: hemorrhage and microbial infiltration. Uncontrolled bleeding is responsible for approximately 20–34% of trauma-related deaths and remains a leading cause of preventable mortality [2]. Concurrently, persistent infections during the healing process can stall the natural recovery cycle, leading to chronic inflammation and severe clinical complications [3]. Under optimal conditions, the body initiates a complex self-repair sequence involving four overlapping stages: hemostasis, inflammation, proliferation, and remodeling, all orchestrated by intricate intercellular signaling [4]. However, when this process is disrupted, it can lead to pathological outcomes, including impaired vascularization, dysregulated growth factor expression, and immune cell dysfunction [5]. Therefore, the primary objective of modern wound care is to provide a multifunctional environment that prevents internal bleeding, blocks external pathogens, and accelerates tissue regeneration.

Current therapeutic strategies for wound management include a range of products, including local hemostats, tissue adhesives, sealants, and traditional dressings such as gauze or bandages. These approaches generally function by either accelerating the coagulation cascade to control hemorrhage [6] or by providing a physical seal for ruptured vessels. Despite their widespread use, many conventional treatments possess significant drawbacks. For instance, certain hemostatic agents can trigger hypersensitivity or allergic reactions [7], and they often lack sufficient biodegradability or biocompatibility. Furthermore, synthetic adhesives designed for high mechanical strength may release toxic degradation products, such as formaldehyde, posing safety risks to surrounding tissues [8,9,10]. While traditional gauze is highly absorbent, its tendency to adhere to the wound bed often causes secondary trauma and additional blood loss during dressing changes.

In this context, biopolymer-based hydrogels have emerged as a highly promising alternative. Hydrogels are three-dimensional networks formed through the physical or chemical crosslinking of hydrophilic polymer chains (hydrogen bonding [11,12], ionic bonding [13,14], or covalent bonding [15,16]). Their high water content and porous structure closely mimic the natural extracellular matrix (ECM), providing an ideal structural scaffold for cellular migration and tissue growth [17]. These materials can effectively donate or absorb moisture to maintain an optimal hydrated environment, which helps soothe the injured site and reduce localized temperature [18,19,20]. Beyond passive protection, hydrogels can serve as active delivery vehicles for antimicrobial agents or growth factors, facilitating their permeation into the wound site and thereby enhancing healing rates [21,22,23]. Their inherent flexibility and conformability allow them to adapt to complex wound geometries, ensuring consistent contact with the skin surface [24]. Moreover, specialized hydrogels with wet-adhesive properties enable immediate attachment to bleeding sites, providing rapid hemostatic support [25,26,27].

Our bodies possess electrical conductivity, and when the skin is injured, the signal transmission between epithelial cells is disrupted [28]. In this scenario, the wound edge becomes the anode, and the central region becomes the cathode, generating an endogenous electric field that stimulates the movement and proliferation of fibroblasts [29,30]. Electrically conductive hydrogels promote this mechanism, enhancing antimicrobial activity and enabling controlled drug delivery [31]. They aid in restoring skin conductivity and in assisting skin fibroblasts in migrating towards the wounded area [32,33]. Electrically conductive hydrogels can also serve as wearable sensors capable of detecting body movements and subtle physiological signal changes [34], enabling real-time monitoring of wound healing [35,36].

Three-dimensional (3D) printing is an attractive technology for personalized medicine and pharmaceuticals, enabling the easy fabrication of intricate and durable structures [37]. Hydrogels, serving as bioinks, can be used with 3D printers to create three-dimensional structures with specific functionalities [38,39]. Various substances, cells, and physiologically active compounds can be combined as needed, enabling precise biomimicry [40]. However, developing effective crosslinking strategies for 3D printed hydrogels to achieve excellent mechanical properties and elasticity remains a significant challenge [41,42].

While several recent review papers have independently addressed standard hydrogel dressings, flexible electronics, or standalone 3D printing methods, this review provides a distinct and timely contribution by focusing specifically on the cross-disciplinary convergence of these fields. Unlike existing literature, we offer a critical synthesis of how tunable electroconductivity and precision 3D architecture directly interface with AI-driven, closed-loop “sense-and-treat” wireless methodologies, mapping out a comprehensive translational roadmap for intelligent personal healthcare.

## 2. Wound Healing Mechanism

**Figure 1:**
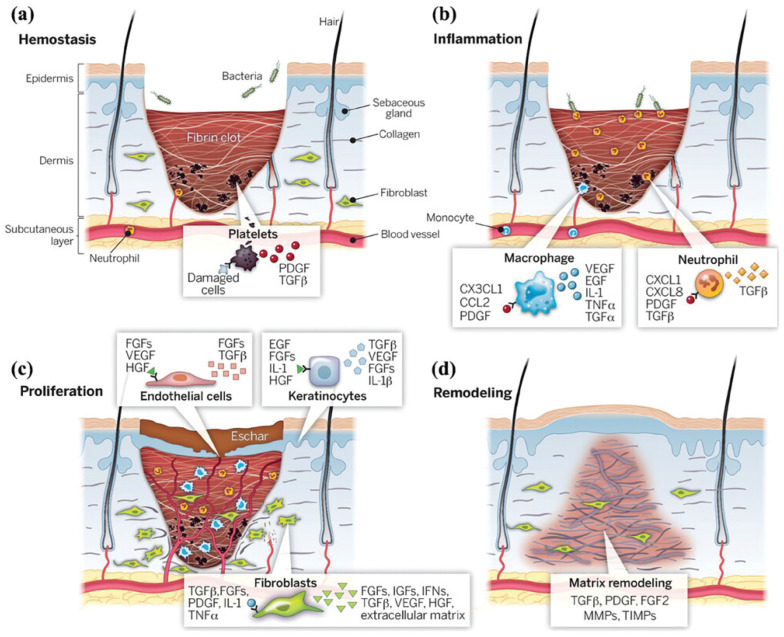
Stages of wound healing [64]. (**a**) Hemostasis. (**b**) Inflammation. (**c**) Proliferation. (**d**) Remodeling.

The healing process involves complex, overlapping stages to restore the structure and function of damaged tissues. These stages can be divided into four interrelated phases: hemostasis, inflammation, proliferation, and remodeling (Figure 1). These phases occur temporally in a complex, interdependent sequence, constituting a sophisticated biological process with feedback mechanisms [43]. Skin wounds generally bleed shortly after occurrence. Simultaneously, blood vessels contract to slow blood flow, reducing blood loss, while platelets attach to exposed subcutaneous tissue via glycoprotein [44]. Activated platelets activate other platelets in the bloodstream to induce clotting and release thromboxane A2 (TXA2) and 5-hydroxytryptamine (5-HT) to reinforce vascular constriction [43], promoting thrombin generation to induce thrombosis [45]. Thrombin is crucial as a procoagulant during the blood clotting stage [45]. Activated thrombin converts fibrinogen to fibrin monomers and promotes clotting, thereby aiding hemostasis [46,47]. Fibrin clotting acts as a scaffold for migrating inflammatory cells (neutrophils, monocytes, etc.), fibroblasts, and endothelial cells, providing a barrier against invading pathogens [48]. Inflammatory cells gathered at the wound site differentiate into macrophages [49], triggering an inflammatory response to remove debris, bacteria, and damaged tissue, and secreting chemokines [50], IL-10, and TGF-β [51], among other growth factors [49,50]. In the innate immune system, macrophages primarily consist of two distinct phenotypes: M1 macrophages, which clear cellular waste and stimulate angiogenesis, and M2 macrophages, which orchestrate tissue repair and regeneration [52]. During the wound healing process, these cells regulate the transition from the inflammatory phase to the tissue remodeling stage by inducing M1/M2 polarization via an adenosine triphosphate (ATP)-mediated promotional mechanism within the wound microenvironment [53]. Concurrently, if M1 macrophages remain abnormally hyperactive over an extended period, the inflammatory response becomes excessively prolonged, potentially progressing into a chronic wound. For this reason, actively modulating the dynamics of M1/M2 macrophage polarization represents a crucial therapeutic strategy for preventing chronic wound formation [54]. In addition, macrophages induce processes such as angiogenesis [55], granulation tissue formation, re-epithelialization [56,57], and wound contraction [58]. Induced fibroblasts [59] and keratinocytes [60] differentiate to form new ECM and remodel by connecting cells [61,62]. During this process, appropriate reactive oxygen species (ROS) provide feedback by inducing cell death to halt granulation tissue formation, thereby regulating the progression of remodeling [63].

## 3. Advanced Multifunctional Capabilities of Hydrogel Dressings

When applied to wound healing, hydrogels exhibit several key advantages: they maintain a moist environment at the wound site, absorb excessive tissue exudates, provide superior oxygen permeability, and facilitate the controlled release of various bioactive factors [65]. Furthermore, multifunctional hydrogel dressings represent an evolutionary step beyond conventional “one-dimensional” treatments that merely provide physical coverage. These advanced materials synergistically combine multiple essential properties (such as sterilization, efficient absorption, and active promotion of tissue regeneration) to accelerate recovery [66]. The specific selection of polymers, nanomaterials, and bioactive agents during the fabrication process significantly dictates the final performance of these dressings. Moreover, these integrated components enable hydrogels to respond to specific physiological stimuli, allowing for sophisticated wound management and real-time monitoring [67].

### 3.1. Hemostatic Performance and Wet-Tissue Adhesion

**Figure 2:**
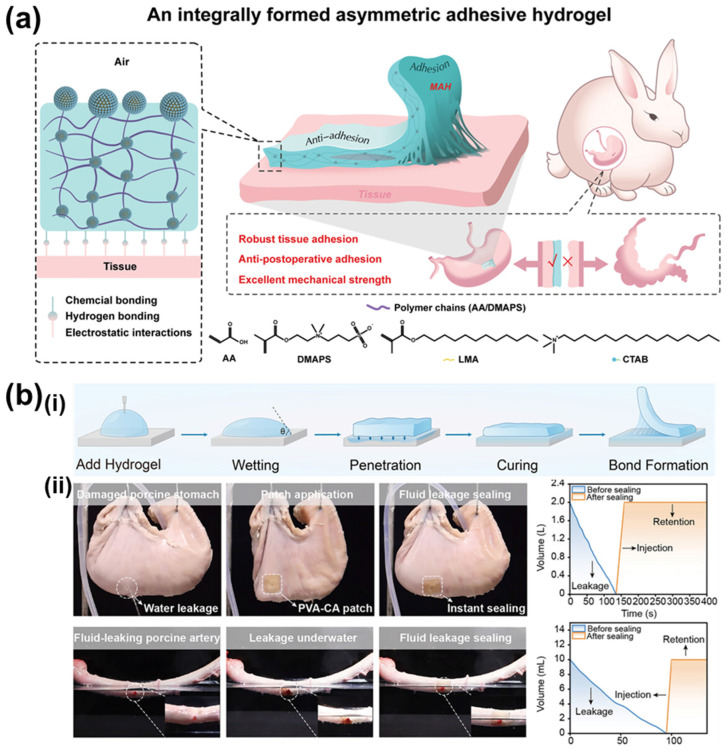
Wet adhesive hydrogels. (**a**) Schematic of the application of wound healing and postoperative adhesion prevention [85]. (**b**) Moist adhesive hydrogel inspired by mussels [86]. (**i**) Schematic of the tissue attachment process. (**ii**) Wet adhesive hydrogel tape for sealing of a fluid-leaking porcine artery and porcine small intestine.

Wet-tissue adhesion is a critical property for maintaining tissue integrity and suppressing localized inflammation during the healing process, especially at interfaces where biological fluids such as blood or sweat are actively secreted [68]. Furthermore, the importance of this moisture-resistant adhesion is even more pronounced in environments that are simultaneously humid, dynamic, and irregular, such as the gingival mucosa. Managing inflammation in these areas is particularly challenging, as it requires high-performance network designs that can provide robust adhesion, extended retention time, and effective antimicrobial activity [69]. When considering internal applications, these challenges become even more significant. Due to the continuously dynamic physiological environment and the frequent contact between different organs, the design must not only ensure adhesion to wet tissue defects but also incorporate anti-adhesive properties on the external surface; consequently, the strategic design of the adhesion mechanism is of paramount importance (Figure 2a) [70,71]. Among various wet adhesion mechanisms, mussel foot proteins (Mfps) serve as a representative biomimetic model [72,73]. Dihydroxyphenylalanine (DOPA), a key constituent of Mfps, plays a pivotal role in wet-tissue adhesion through diverse interactions, including hydrophobic effects and coordination bonding [74]. Beyond these chemical functions, mussels achieve wet adhesion through spatially organized interactions, in which the adhesive strength is determined by the specific number and arrangement of functional groups [75,76]. In one study, they developed a hydrogel with tunable cohesion and adhesion achieved by modulating the microenvironment. This hydrogel, synthesized via the interaction of DOPA-derived catechol derivatives with polyvinyl alcohol (PVA) and caffeic acid, demonstrated the fundamental stages of effective wet-tissue adhesion: wetting, adsorption, penetration, curing, and stable bond formation (Figure 2b(i)). The clinical potential of this system was confirmed when applied to the stomach and small intestine, where the hydrogel was shown to halt the leakage of internal fluids immediately (Figure 2b(ii)). As an alternative strategy for wet adhesion, another approach involves removing the hydration layer at the tissue interface by absorbing interfacial water. This method, utilizing powder-based hydrogels, ensures stable and intimate contact with tissues, allowing for effective adhesion even at irregularly shaped wound sites [77,78]. In one study, they developed a wet-tissue adhesive powder hydrogel using highly modified polymers. They compared its performance with that of conventional pre-gel and in situ gel systems [79]. The powder hydrogel demonstrated a high adhesive strength of 7.5–15.2 kPa, significantly outperforming commercial fibrin glue, which recorded only 2.8 kPa. Notably, the powder hydrogel maintained stable adhesion and provided effective hemostasis in actively bleeding areas where other hydrogel forms typically fail to adhere. While these biochemical and physical capabilities are crucial for initial wound stabilization, integrating electrical conductivity into hydrogels can further accelerate wound healing by stimulating endogenous electric fields (EFs). Generally, endogenous EFs establish a transepithelial potential difference arising from the asymmetric distribution of ion channels (specifically cations (Na^+^) and anions (Cl^−^)) in epithelial cells. Upon injury, an electric field is generated across the damaged area, characterized by a cathode at the center and anodes at the margins, which serves as an essential mechanism for re-epithelialization [30]. Consequently, applying an artificial electrical stimulation (ES) that mimics this natural transepithelial potential (10–60 mV) facilitates cellular homeostasis, promotes cell migration, proliferation, and differentiation, enhances angiogenesis, and modulates inflammatory responses, thereby accelerating the overall wound healing process [80,81]. Particularly, within a 3D microenvironment like a hydrogel, such stimulation can effectively guide cell spreading, orientation, and targeted migration [82]. Because this approach offers the distinct advantage of precisely controlling complex tissue regeneration, it ultimately underscores the necessity of developing patient-tailored, conductive 3D-modeled hydrogels for complex and irregular wound sites [83,84].

### 3.2. Antimicrobial and Anti-Inflammatory Properties

**Figure 3:**
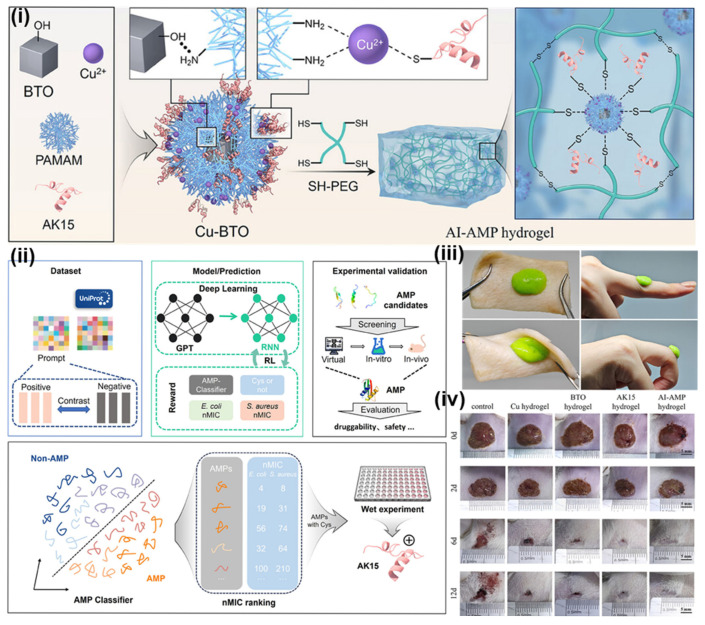
Antimicrobial and anti-inflammatory hydrogels [118]. (**i**) Schematic of the AI-AMP hydrogel design. (**ii**) Schematic of AI mechanisms to distinguish ATM. (**iii**) Image of the biocompatibility of hydrogels. (**iv**) Image of the rate of wound healing depending on the material.

Antimicrobial activity is indispensable for the prevention and treatment of various infectious diseases. Since the introduction of penicillin, antibiotics have served as the primary defense against bacterial infections [87]. Hydrogels are highly effective candidates for antimicrobial applications due to their unique properties, including high swelling capacity [88,89], oxygen permeability [90], biocompatibility [91], and the ease of drug loading and release [92,93]. Hydrogels can enhance their antimicrobial efficacy by incorporating antimicrobial agents, metal nanoparticles [94,95], or polymers with inherent antibacterial activity. In particular, hydrogels based on natural materials (such as chitosan and antimicrobial peptides (AMPs) [96,97]) are widely used in wound dressing research because they avoid the biocompatibility limitations often associated with synthetic materials. In one study, researchers fabricated an antimicrobial hydrogel by combining a self-powering material with the AK15 peptide, which exhibits strong activity against *Escherichia coli (E. coli)* and *Staphylococcus aureus (S. aureus)* (Figure 3(i)). The selection of AK15 was facilitated by artificial intelligence (AI). Specifically, the platform used a large language model (LLM) pre-trained (PT) on an extensive collection of existing peptide data, which was subsequently task-specifically PT using an AMPs dataset. To further optimize the PT model, reinforcement learning (RL) was implemented, through which a customized reward function was formulated to specifically guide the design of AMPs targeting *E. coli* and *S. aureus*. Within this framework, Marcel—a specialized AMPs predictor—was employed as the reward function to evaluate antimicrobial activity and predict the normalized minimum inhibitory concentration (MIC) values against *E. coli* and *S. aureus*. Finally, cysteine residues were strategically incorporated into the peptide sequences to enable cross-linking, thereby ensuring sustained and long-term antimicrobial efficacy (Figure 3(ii)). This antimicrobial hydrogel maintained stable adhesion during bodily movements (Figure 3(iii)) and demonstrated complete healing of infected neck wounds in rat models within six days (Figure 3(iv)).

The prolonged use and misuse of antibiotics have led to the emergence of antibiotic-resistant bacteria [98,99], creating a concerning cycle in which increasingly potent antibiotics [100,101,102] are required to combat resistance. In this case, going beyond passive drug diffusion, electroconductive hydrogels offer sophisticated auxiliary antibacterial pathways. Characterized by excellent biocompatibility and design flexibility, hydrogels can precisely modulate drug release profiles in real time, dynamically responding to both endogenous factors (e.g., pH, ROS, and specific biomolecules) and exogenous stimuli (e.g., light, ultrasound, and electricity) [103,104,105,106]. Recently, electronically controlled drug delivery systems have emerged as highly attractive platforms owing to their rapid responsiveness, straightforward operation, and exceptional spatiotemporal resolution [107,108,109]. ES allows for the fine-tuning of drug release rates by adjusting specific parameters, including current waveform, intensity, frequency, and duration. When integrated into a hydrogel matrix that possesses robust tissue adhesiveness and high drug-loading capacity, ES offers active temporal control over drug release profiles by leveraging the redox transitions of the conductive network [110]. In one study, a biocompatible electronic wound-healing patch was developed by integrating advanced strategies encompassing both ES mode engineering and electrode configuration design [111]. The resulting hydrogel, engineered as an electro-responsive matrix and drug reservoir, exhibited an exceptionally high drug-loading capacity of up to 90% alongside outstanding mechanical resilience. Furthermore, by systematically optimizing key ES parameters (e.g., voltage, frequency, and waveform) the authors demonstrated that the conductive hydrogel platform enables precise spatiotemporal control of drug release.

Inflammation is an essential phase of wound healing, serving as a primary response to remove foreign bodies, pathogens, and necrotic debris while providing a microenvironment for tissue regeneration [112]. Nevertheless, persistent inflammation is a leading cause of chronic wounds, as endogenous or exogenous factors can disrupt inflammatory regulation and impede healing [113]. Because excessive immune activity can lead to the destruction of healthy tissue, sophisticated anti-inflammatory strategies are required to optimize the inflammatory response [114,115]. Bioactive hydrogels support stable healing by incorporating biomaterials with immunomodulatory properties, such as those that promote macrophage polarization [113,116]. Researchers fabricated an immunomodulatory hydrogel using gallic acid (GA)-modified chitosan (CS-G) and dopamine (DOPA)-modified silk fibroin (SF-D) to regulate M2 macrophages and establish a regenerative protein microenvironment. To induce the M1 phenotype and facilitate hydrogel crosslinking, ethanol (EtOH) and sodium periodate (NaIO_4_) were utilized [117]. The antimicrobial effect of this hydrogel increased proportionally with the EtOH concentration. Regarding cytotoxicity, while EtOH initially inhibited cell growth on Day 1, cell proliferation significantly increased in the EtOH-treated groups once the medium was replaced and the EtOH was removed, demonstrating that cellular behavior can be controlled over time. Similarly, while M2 polarization was initially suppressed in the EtOH-containing hydrogels on Day 1, it reached its peak by Day 4, confirming that macrophage polarization can be sequentially and precisely induced.

### 3.3. Tailored Hydrogel Designs for Specific Wound Microenvironments

Cutaneous tissue damage arises from a multitude of pathological factors (including diabetes, thermal burns, and persistent infections) rather than simple trauma. Consequently, instead of employing a standardized, one-size-fits-all wound care approach, it is imperative to deeply understand the specific wound microenvironment and establish corresponding targeted therapeutic strategies [119]. Diabetes, a metabolic disorder characterized by deficient insulin secretion, leads to chronically elevated blood glucose levels, which subsequently cause severely impaired wound healing. Unlike conventional acute wounds, diabetic wounds are classified as chronic wounds, demanding advanced solutions capable of addressing a complex microenvironment (characterized by hyperglycemia and low pH) as well as exceptionally high recurrence rates [120,121]. In one study, hydrogels were fabricated by strategically introducing tannic acid (TA) into a chitosan/poly(vinyl alcohol) (CS/PVA) matrix, thereby presenting a robust and highly efficient drug delivery platform [122]. The hydrogel, which possessed a highly porous structure, incorporated FG4592 (or roxadustat) (prolyl-4-hydroxylase inhibitor) to stabilize hypoxia-inducible factor-1α (HIF-1α). This approach effectively accelerated the treatment of anemia in patients with chronic diabetic complications and confirmed the expanded therapeutic scope of the platform, including the stimulation of angiogenesis and the activation of stem cells. Concurrently, the hydrogel adhered firmly to the tissue substrate, mitigating oxidative stress, modulating inflammatory responses, and promoting robust angiogenesis, thereby proving its capacity to resolve complex physiological and pathological cascades.

For burn wounds, emergency intervention is often severely hindered by hypersensitive pain, irregular lesion shapes, massive exudate production, and high susceptibility to secondary infections [123,124]. Furthermore, the absence of immediate and proper first aid carries the risk of severe pain, extensive tissue necrosis, and permanent disability, which underscores the urgent need for an advanced healing platform that provides concurrent immunomodulation and thermal regulation [125,126]. In one study, an ionic/electronic conductive hydrogel was fabricated using sodium alginate (SA), calcium ions, and poly(3,4-ethylenedioxythiophene):poly(styrenesulfonate) (PEDOT:PSS)), which exhibited excellent conformal adaptability to irregular wound topologies upon injection [127]. This hydrogel achieved a skin-like electrical conductivity (0.0026 S/cm) while demonstrating remarkable dynamic viscoelasticity and mechanical stability capable of withstanding physiological stress (compressive stress: 21.3 ± 1.5 kPa, elastic modulus: 20.8 ± 1.8 kPa). Upon the application of ES, the platform significantly accelerated the overall wound healing process by enhancing angiogenesis, driving macrophage polarization, and reinforcing epithelial regeneration.

Infectious wounds represent another prominent class of chronic wounds, where continuous bacterial colonization disrupts tissue repair mechanisms and exacerbates persistent inflammatory states; these pathogens actively resist the host immune system and conventional antimicrobials, further aggravating the infection and stalling the recovery timeline [128,129]. Owing to their inherent water retention capacity, high gas permeability, and excellent biocompatibility, hydrogels can provide an ideal moist yet controlled microenvironment for managing wounds prone to chronicity. Moreover, leveraging their stimuli-responsive properties allows for the controlled release of antimicrobial agents, facilitating precise infection management and accelerated wound healing [130]. A Cuf-MET hydrogel was fabricated by utilizing metformin (MET) to induce the fibrillation of copper formate (Cuf) in an aqueous solution [131]. By precisely regulating the release kinetics of copper ions, the hydrogel exerted exceptional antimicrobial activity within a non-toxic concentration window, achieving an efficiency of 99.82% ± 0.47%, against *E. coli* and 98.98% ± 1.25% against *S. aureus*. Furthermore, the Cuf-MET hydrogel demonstrated a distinct antifungal effect by eradicating biofilms through electrostatic interactions with fungal cells, confirming its capacity to systematically disrupt the structural integrity of the fungal cell wall and cytoplasmic membrane.

## 4. Electrical Conductivity Materials for Bioactive Scaffolds

Due to their inherent biocompatibility, high water content, and ability to mimic ECM, stimuli-responsive hydrogels are ideally suited for monitoring specific biological sites [132]. By integrating electrical conductivity into these systems, the real-time monitoring of physiological changes and numerical data becomes more accessible, thereby facilitating timely therapeutic interventions. In particular, these conductive systems are widely utilized in specialized fields such as targeted drug delivery [133,134] and tissue regeneration [135]. Conductive hydrogel sensors simultaneously exhibit stimuli-responsiveness and electrical conductivity—triggered by physicochemical or biochemical stimuli—through the incorporation of conductive polymers (CPs) (e.g., polyaniline (PANi), polypyrrole (PPy), and PEDOT:PSS, carbon-based nanomaterials (e.g., carbon nanotubes, graphene, and carbon dots), metal nanoparticles (MNPs) (e.g., gold nanoparticles (AuNPs), silver nanoparticles (AgNPs), and copper nanoparticles (CuNPs)), or self-powered materials [136]. Table 1 provides a comprehensive summary and classification of existing conductive hydrogel sensors. As demonstrated in the literature, hydrogels can detect signals through diverse mechanisms depending on the sensor type, enabling highly tailored applications in clinical settings.

**Table 1:** Summary and classification of conductive hydrogels for tissue engineering.

Hydrogel Composition	Cross-Linking	Conductivity	3D Printing	Application	Ref.
Carboxymethyl chitosan (CMC), Oxidized sodium alginate (OSA), polymerized GA, Fe^3+^	Imine bond, Organic–metal complexes	1 × 10^−5^~0.26 S/m	N/A	Antibacterial activity, macrophage polarization, and upregulation of collagen synthesis (TGF-β) and angiogenic factors (CD31)	[137]
Poly(acrylic acid), poly(ethylenimine) (PEI), silver nanoparticles, PPy, Co^2+^	Radical polymerization, hydrogen bond	0.048 S/m	N/A	Promotion of antibacterial activity, angiogenesis, and diabetic ulcer healing	[138]
Chitosan (CS), TA, PAA, sulfobetaine methacrylate (SBMA), Al^3+^	Metal ionic coordination, hydrogen bond	3.8 S/m	N/A	Antibacterial and anti-inflammatory	[139]
GelMA, CS, PPy	Photopolymerization	797 S/cm	Extrusion	Peripheral nerve injury repair	[140]
PVA, κ-carrageenan, Catechin-loaded mesoporous ZnO, PEDOT:PSS	Radical polymerization	0.532 S/m	Extrusion	Antibacterial activity, blood coagulation, and acceleration of wound healing	[141]
SA, Gelatin, potassium chloride, gallium-based liquid metal	Ionic bond	1.36 S/m	Extrusion	Antibacterial activity and cell proliferation	[142]
Acrylamide, polyethylene glycol diacrylate (PEGDA), MXene	Photopolymerization	N/A	Digital light processing (DLP)	Promotion of frostbite wound healing	[143]
Gelatin methacryloyl (GelMA), CS, PEDOT	Photopolymerization	0.18 S/m	DLP	Peripheral nerve injury repair	[144]

### 4.1. Conductive Polymers

CPs are defined as polymers in which electrons are loosely bound to the polymer chains. Specifically, certain polymers possess a conjugated backbone structure characterized by strong π-orbital overlap; upon doping, these systems exhibit high electrical conductivity [145]. Among these materials, those with excellent biocompatibility can be used to stimulate cultured cells or tissues by delivering electrical signals. However, their inherent mechanical brittleness and poor processability often limit their practical applications. To overcome these limitations, CPs are frequently used in tissue engineering as conductive composite hydrogels, which are further blended with other materials to enhance mechanical strength [127]. In contrast, CPs have received significant attention as electroactive hydrogels due to their high conductivity (10^−3^–10^5^ S/cm) and their ability to overcome the limitations of conventional polymers through low solubility and tissue-adhesive properties. Achieving complete in vivo biodegradation remains a critical challenge [146].

PANi is a material that can be synthesized through the chemical or electrochemical oxidation of aniline monomers in acidic solutions [147]. The repeating skeletal structure of aniline in PANi allows for the tuning of its electrical conductivity, which is influenced by the redox state of the nitrogen atoms between the phenyl rings, the degree of protonation, the type and concentration of dopants, and the temperature; consequently, various synthesis methods, including chemical and electrochemical oxidation, have been extensively studied [148]. PANi exhibits excellent stability, cost-effectiveness, and easily adjustable electrical conductivity. Furthermore, when utilized in scaffold configurations, it demonstrates the ability to prevent microbial infections, making it a preferred choice for applications such as antibacterial drug delivery systems and biosensors [149,150]. However, PANi faces challenges of low processability, limited flexibility, and concerns regarding biodegradability and cytotoxicity, which necessitate further countermeasures [151]. In addition, PANi, due to its low solubility in organic solvents and inherent brittleness caused by rigid π-conjugated bonds, remains difficult to fabricate complex geometries even when synthesized or integrated with highly biocompatible polymers [152]. In one study, to overcome the limitations of PANi, a GelMA-PANi composite was synthesized using GelMA. The GelMA-PANi composite hydrogel exhibited mechanical properties (12.62 ± 0.65 kPa) and cell adhesion characteristics similar to those of biomimetic GelMA, while also demonstrating excellent performance in producing specific microstructures using stereolithography. In another study, PANi was fabricated into nanoparticles (NPs) via a multiphase synthesis method, allowing it to withstand significant strain and stress, unlike conventional PANi fibers, which are inherently brittle [153]. By utilizing these PANi-NPs as conductive fillers and crosslinking agents, the interactions within the polymer matrices were enhanced through non-covalent bonds, such as hydrogen bonding and electrostatic interactions. This approach resulted in remarkable stretchability (≈1000%), ultra-softness (≈6 kPa), and a high self-healing efficiency (93.3%) at room temperature, demonstrating the potential for wearable patches capable of sensing human motion and external stimuli.

PPy can be prepared by electrochemical polymerization of pyrrole or by oxidation of pyrrole with sulfuric acid [154]. The resulting PPy is inherently insoluble and mechanically rigid, which tends to reduce the overall mechanical performance of polymer compositions [155,156]. For this reason, PPy is typically dispersed between biomaterials or bonded to them via in situ polymerization to impart conductivity to the polymer network [157,158]. PPy was uniformly loaded onto an anisotropic bamboo template (ABT)—a natural layered structure of bamboo modified with lignin—and a hydrogel was fabricated by introducing flexible polyacrylamide (PAM) [159]. This hydrogel functioned as a robust supercapacitor, exhibiting high mechanical strength (104.82 MPa) and high capacitance (1377 mF/cm^2^). Another study established a uniform conductive system by conjugating PPy with gelatin to create gelatin-PPy, in which the insolubility of PPy and the non-conductivity of gelatin were complementary [160]. As the gelatin-PPy content increased, both the conductivity (1.63 × 10^−4^ S/cm to 5.52 × 10^−4^ S/cm) and the elastic modulus (11.9 kPa to 20.77 kPa) showed a corresponding increase. These values align with the electrophysiological regulation of cardiac tissue and the compressive elasticity range of the myocardium (11.9–46.2 kPa [161]). Notably, when applied to myocardial conduction symptoms, it was confirmed that electrical signals were efficiently delivered to the myocardial infarction site.

PEDOT:PSS is a composite where the conductive polymer PEDOT—which is resistant to decomposition by oxygen and water and possesses electronic conductivity but is insoluble—is mixed with the electrolyte PSS. PSS ensures water solubility and stability, stabilizes the doped PEDOT, and provides a matrix for easy dispersion in solvents [162,163]. PEDOT:PSS formed in this manner is an attractive material due to its easy processability, high stability at room temperature, and ability to implement various levels of conductivity while maintaining transparency in the visible light region [164]. In one study, PEDOT:PSS was combined with SA to form a hydrogel with skin-like conductivity (0.0026 S/cm), serving as a dressing that promotes wound healing [127]. This hydrogel exhibited enhanced elastic modulus (20.8 ± 1.8 kPa) and self-healing properties; when ES was applied after attachment to the wound site, it achieved a wound closure rate of 96.46 ± 0.14% within 10 days. During this process, the hydrogel demonstrated excellent adhesion and adaptability to the wound environment and accelerated various healing mechanisms, including promoting angiogenesis and regulating macrophage polarization. In another study, a reusable hydrogel was fabricated by introducing PEDOT:PSS and polydopamine (PDA), a dynamic non-covalent bond enhancer, into PAM [165]. This hydrogel exhibited high conductivity (5.57 S/m) and a Young’s modulus (2.7 kPa) similar to skin tissue. It also demonstrated an adhesive strength of 46.45 ± 1.75 kPa when attached to porcine skin, confirming its capability as a wearable patch. Furthermore, it showed high accuracy and convenience for electromyography (EMG) monitoring, with a high signal-to-noise ratio of 30.05 dB.

### 4.2. Carbon-Based Nanomaterials

Carbon-based nanomaterials (CBNs) refer to nanomaterials that exhibit various arrangements and characteristics based on combinations of sp^2^ and sp^3^ hybridized carbon bonds, morphologies, and functional groups [166]. CBNs can adopt dimensional structures tailored to specific needs, such as zero-dimensional (0D) carbon dots (CDs), one-dimensional (1D) carbon nanotubes (CNTs), and two-dimensional (2D) graphene. CBNs exhibit high mechanical strength, high electrical conductivity, and excellent biocompatibility; due to these physicochemical properties, they have been widely utilized in scaffolds for tissue engineering applications. When CBNs are incorporated into hydrogel networks, they minimize mechanical discrepancies, maintain biocompatibility during mixing, and provide electrical conductivity proportional to their content, thereby imparting sensitivity to electrical signals as required by the specific context [167].

**Figure 4:**
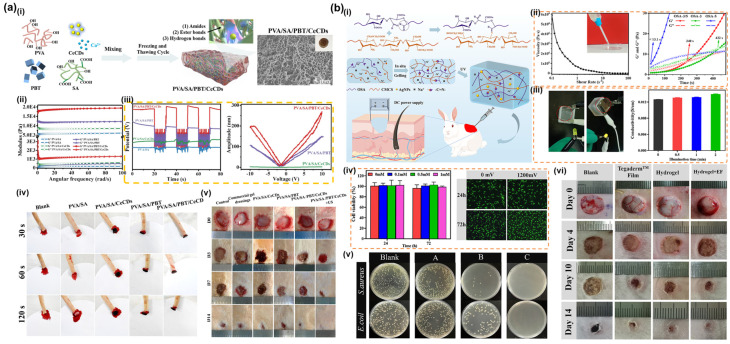
Electrically conductive hydrogels. (**a**) Electrically conductive hydrogels with carbon-based nanomaterials [187]. (**i**) Schematic and structural image of a PVA/SA/PBT/CeCDs hydrogel incorporating a piezoelectric material (PDA−coated barium titanate (PBT)) and CeCDs. (**ii**) Rheological graphs in an angular frequency environment with and without nanomaterials. (**iii**) Open-circuit voltage and butterfly-shaped amplitude-voltage curves according to the presence of nanomaterials. (**iv**) Photographs of hemostasis progress in a tail vein bleeding model. (**v**) Photographs of wound closure rate progress in an infected wound model. (**b**) Electrically conductive hydrogels with metal nanoparticles [188]. (**i**) Schematic of the fabrication and application of an OSA/CMCS/AgNPs hydrogel with in situ synthesized AgNPs. (**ii**) Rheological property analysis of OSA/CMCS/AgNPs. (**iii**) Conductivity analysis according to AgNPs content. (**iv**) Biocompatibility analysis according to AgNPs content and electrical activation. (**v**) In vivo evaluation of the hydrogel for wound healing. (**vi**) Antibacterial evaluation according to AgNPs content.

CDs are promising materials for biological applications due to their inherent band gap, excellent grafting ability, low production costs, and dispersibility in various solvents [168]. Furthermore, CDs are luminescent carbonaceous nanoparticles that can be endowed with chiral properties; they are promising for biomedical applications due to their low cytotoxicity and ease of synthesis [169]. As carbon-based nanomaterials with nanoscale dimensions, quasi-spherical shapes, excellent biocompatibility, high quantum yields, and strong absorption, CDs possess fascinating optical features, including ultraviolet (UV) absorption, photoluminescence, up-conversion fluorescence, and long-wavelength/multicolor emission [170]. Additionally, their surfaces are composed of functional groups such as amino, carboxyl, and aldehyde groups, allowing for the improvement or addition of new functionalities, such as water solubility and antibacterial properties [171]. These surface functional groups play a crucial role in the construction of scaffold structural units. Under the synergistic effect of non-covalent interactions and covalent bonding, CDs serve as central nodes for dense crosslinking with various natural polymers (such as chitosan, cellulose, agarose, and DNA) during scaffold fabrication [172]. In one study, a responsive and conductive hydrogel was fabricated that released CDs and Mg^2+^ in response to pH to scavenge ROS and promote macrophage polarization [173]. When this hydrogel was applied to a diabetic chronic limb ischemia (CLI) environment, it was confirmed to inhibit stem cell apoptosis and promote nerve and vascular repair. Another study developed CeCDs by doping CDs with cerium to enhance network interactions within the hydrogel. This resulted in an intelligent wound-healing hydrogel that increased the piezoelectric output of the piezoelectric hydrogel and mitigated oxidative stress (Figure 4a(i)). The introduction of piezoelectric materials and CeCDs was shown to increase the rheological behavior, voltage output, and piezoelectric response of the hydrogel, proving that these nanomaterials contributed to network reinforcement (Figure 4a(ii,iii)). In clinical experiments, the addition of these nanomaterials shortened rat tail vein hemostasis from 230 s to 44 s (Figure 4a(iv)), and in an infected rat wound model, a closure rate of 93.8% was observed by day 14 (Figure 4a(v)).

CNTs possess a seamless hollow cylindrical structure. They are referred to as single-walled carbon nanotubes (SWCNTs) when a single-atom-thick graphite sheet is rolled into a tube, and multi-walled carbon nanotubes (MWCNTs) when multiple layers form the tube [174]. CNTs exhibit superior mechanical strength, with tensile strengths of 11–52 GPa and bending strengths of 14.2 ± 8 GPa, making them excellent candidates for reinforcing agents and for imparting conductivity to hydrogels [175]. However, CNTs can exhibit cytotoxicity beyond mere antibacterial properties and tend to aggregate due to Van der Waals forces, leading to low solubility in solvents; thus, these issues must be addressed through chemical modification [176,177]. In one study, carboxylated CNTs were incorporated into a cationic hydrogel (P(AM-co-DMDAAC), PAD) to enhance the stretchability and conductivity of the non-ionic conductive hydrogel via electrostatic interactions, hydrogen bonding, and entanglement [178]. After dehydration, the conductivity of this hydrogel was confirmed to range from 2.3 S/m to 25 S/m, depending on its density. This demonstrates its potential applications for swelling response, variable resistance, and strain sensors. In another study, CNTs were incorporated into GelMA using electrospinning to produce aligned conductive hydrogel fibers [179]. In vitro studies showed that these hydrogel fibers promoted cell proliferation and aligned adhesion through electrical stimulation. When implanted into an injury model, the CNTs remained at the injury site while the GelMA fibers biodegraded, continuously enhancing the conductivity of the regenerated tissue. Throughout this process, even though the CNTs did not biodegrade, no diffusion or toxicity was observed, and their ability to regulate inflammation and induce nerve fiber regeneration was confirmed.

Graphene is the fundamental structure of graphite, consisting of a single layer of carbon atoms, and is a key element encompassing various derivatives with diverse forms and properties [180]. A representative example is graphene oxide (GO), which is synthesized by oxidizing graphite and possesses excellent water solubility, amphiphilicity, and ease of surface modification. GO is readily dispersed in solvents due to its abundant oxygen-containing functional groups and exhibits high compatibility and reactivity with other materials; however, caution is required, as this reactivity can alter biochemical properties [181,182]. In one study, GO and fibroblast exosomes were introduced into a silk fibroin-based hydrogel to combine electrical properties with biological signals [183]. As a result, the hydrogel was confirmed to not only effectively mimic the neural microenvironment but also effectively promote axonal growth, myelination, and vascular regeneration. In this hydrogel composition, GO played a key role in enhancing electron transfer, which is essential for cellular activity. Reduced graphene oxide (rGO) is produced by reducing GO using a reducing agent. This offers the advantage of being highly convenient for applications, as it allows for easy adjustments in structure, electrical conductivity, hydrophilicity, functional groups, and color [184]. However, if excessive reduction occurs, oxygen and hydrophilic functional groups are nearly eliminated, making it difficult to bond with hydrophilic polymers; thus, care must be taken during the manufacturing process [185]. In one study, rGO was incorporated into a hydrogel composed of SA and gelatin to evaluate swelling behavior, mechanical properties, and anti-fibrotic efficacy at varying concentrations [186]. Furthermore, in addition to ionic crosslinking, rGO enhanced 3D printing stability and provided the necessary stiffness and reduced fibrosis required at the harvest site during urethral reconstruction.

### 4.3. Metal Nanoparticles

MNPs are generally crystalline materials in three dimensions that can impart various physical, chemical, optical, magnetic, and electronic properties to hydrogels, depending on the specific metal used [189]. Furthermore, metal nanoparticles possess a high surface-area-to-volume ratio and can be easily synthesized into various morphologies with high chemical activity [190]. Hydrogels incorporating these MNPs can induce responsiveness to environmental stimuli, and the efficiency of these properties can be enhanced by precisely arranging the particles within the matrix [191,192]. Consequently, these materials play a vital role in sensing responses at defect sites and facilitating mechanisms that promote tissue regeneration [193,194,195].

AuNPs are characterized by their ease of preparation, stability, tunable size, high surface area, and ease of functionalization [196]. In particular, they are more frequently utilized than other MNPs due to their superior electrical conductivity and corrosion resistance [197]. While AuNPs can interact directly with bacterial cells to penetrate cell membranes and induce lysis, strategies to mitigate potential tissue cytotoxicity are required [198,199]. In one study, researchers integrated PEI-coated AuNPs into an oxidized SA hydrogel to create a platform that simultaneously promotes tissue regeneration and signal transduction [200]. This hydrogel was confirmed to effectively deliver drugs to cardiomyocytes, increasing physiological beating rates and contraction amplitudes while inducing rhythmic beating. Additionally, while conductivity and rheological performance improved as the nanoparticle content increased, cell viability was affected when the AuNPs concentration exceeded 5% due to their inherent cytotoxicity. Another study developed a hydrogel patch by adding AuNPs to a carboxymethyl cellulose and acrylamide-based network, which induced strong hydrogen bonding interactions within the polymer. This resulted in excellent mechanical strength (22.8 MPa) and adhesive strength (246 kPa) [201]. Although this hydrogel exhibited high antibacterial activity (98.2% against *E. coli* and 99.1% against *S. aureus*), this research also highlighted the need for countermeasures against potential cytotoxicity as the AuNP content increases.

AgNPs possess a large surface-area-to-volume ratio due to their unique structure, allowing them to adhere to microbial surfaces, increase permeability, and disrupt interactions with cell membrane proteins [202]. Specifically, their uptake mechanism is reported to involve macropinocytosis, scavenger receptor-mediated, and clathrin-mediated pathways, leading to biological effects such as changes in cell morphology, oxidative stress, DNA damage, and subsequent apoptosis [203]. Therefore, cytotoxicity can be modulated by adjusting oxidative stress and the release of Ag^+^ ions from AgNPs dissolution, thereby controlling the stimulation of inflammatory and immune responses [204]. Since this cytotoxicity primarily affects cells containing peptidoglycan, which is absent in mammalian cells, the risk of toxicity during human application is generally considered low [205]. Due to these unique antimicrobial properties, the combination of AgNPs with various polymers and biomaterials significantly enhances both electrical conductivity and antibacterial efficacy [206]. In one study, an OSA/CMCS/AgNPs hydrogel was manufactured via the in situ formation of AgNPs through UV irradiation within an amine-bonded matrix composed of oxidized SA and CMC (OSA/CMC) (Figure 4b(i)). The OSA/CMCS network exhibited shear-thinning properties, making it injectable, and formed a stable crosslinked structure as the oxidation degree of the SA increased (Figure 4b(ii)). Notably, the introduction of AgNPs increased the electrical conductivity from 0.010 S/cm to 0.0127 S/cm, with conductivity rising as longer UV exposure times led to increased AgNPs formation (Figure 4b(iii)). Furthermore, cell proliferation remained above 90% even when the AgNPs concentration was increased to 1 mM, while antibacterial efficiency simultaneously improved (Figure 4b(iv,v)). When applied to an infected skin model in rabbits, this hydrogel showed a high wound-healing rate, which was further accelerated by ES (Figure 4b(vi)).

CuNPs offer attractive features for tissue engineering, including low cost and high clinical safety [84]. CuNPs influence the production, synthesis, and stabilization of ECM proteins and exhibit excellent antibacterial properties [207,208]. In one study, fibers were fabricated by depositing CuNPs onto bacterial cellulose (BC@Cu) and then introduced into a network composed of SBMA and acrylamide to create a hydrogel with superior antibacterial and conductive properties [209]. The introduction of BC@Cu increased the compressive strength from 0.09 MPa to 0.61 MPa and the conductivity from 17.23 mS/m to 41.25 mS/m, significantly enhancing both mechanical and electrical characteristics. Regarding biocompatibility, toxicity tests based on Cu^2+^ ion release showed that the concentration reached 3.3 mg/mL after 72 h, maintaining antibacterial activity while remaining within the acceptable range for human biocompatibility. In practice, the hydrogel demonstrated 99% bacterial removal and achieved a high wound recovery rate of 89.3% under an ES environment.

### 4.4. Self-Powered Materials

The integration of self-powering mechanisms into hydrogel platforms has been driven by the increasing need to miniaturize and simplify ES devices [210]. Generally, self-powering is achieved through two primary methods: piezoelectric nanogenerators (PENG) and triboelectric electrostatic nanogenerators (TENG). These technologies enable the hydrogel network to convert mechanical energy into electricity, making it a self-sustaining power source [211]. PENG hydrogels are fabricated by incorporating materials with piezoelectric properties—such as ceramics, single crystals, or specific polymers—into the hydrogel matrix. In their steady state, piezoelectric materials remain neutral because the centers of negative and positive charges within the structure overlap. However, external mechanical stress deforms the internal structure, causing these charge centers to separate and form electric dipoles, thereby creating an unbalanced state. At this stage, a voltage is generated by changes in the polarization direction, and the specific output can be modulated according to the degree of polarization change [212]. Barium titanate (BTO) is a representative piezoelectric ceramic and the most extensively researched material among perovskite (ABX_3_) compounds; its piezoelectric effect stems from the displacement of central ions within its tetragonal or orthorhombic crystal structures, which causes an interfacial imbalance [213]. In one study, researchers integrated BTO into a hyaluronic acid-based hydrogel to create a platform that delivers ES in response to ultrasonic triggers [214]. With the addition of BTO, the hydrogel produced an output voltage of approximately 70 mV under ultrasound; notably, this piezoelectric effect promoted the expression of Col II and SOX9 proteins, as well as the growth of RAW264.7 and BMSC cells. This confirmed that the nanomaterial not only enhances physicochemical properties and biocompatibility but also contributes to antioxidant activity and tissue regeneration via the piezoelectric effect. Nevertheless, because ceramics are inherently brittle, careful consideration regarding their biosafety is required for clinical applications [215]. TENG generates power through a combination of triboelectrification and electrostatic induction. Friction between two materials causes one to lose electrons (becoming positively charged) and the other to gain them (becoming negatively charged), leading to an interfacial charge imbalance and inducing electron flow through a polarization effect to output power [216]. Typically, TENG systems are designed as multilayer structures; by incorporating PENG characteristics into these layers, researchers can achieve higher charge densities and improved power output capacities [217,218,219]. In one study, a thermoelectric neural network skin patch was developed using a molybdenum disulfide (MoS_2_)-based hydrogel and GelMA, integrating self-powering with thermal stimulation [220]. This system achieved a maximum voltage of 48.80 V and a short-circuit current of 0.57 μA. As the external force applied to the hydrogel increased from 1 N to 50 N, the transferred charge density rose from 52.75 nC to 261.5 nC; furthermore, the voltage output increased from 21.38 V to 101.38 V, depending on the thickness. Remarkably, this hydrogel demonstrated a synergistic antibacterial effect against *S. aureus* and *E. coli* when TENG-induced activity was combined with NIR irradiation; it was also observed that the photothermal effect of NIR was significantly enhanced by the TENG properties. When applied to an in vivo wound model, the TENG properties alone resulted in an 82.34% recovery rate, while the synergy with NIR irradiation reached 90.44% recovery within just 5 days.

Although conductive hydrogels can be used to accelerate wound healing owing to their electrical conductivity comparable to that of human skin, their conductivity often varies significantly with diverse environmental factors, such as pH, protonation states, and the wound microenvironment. Consequently, balancing electrical conductivity with biological safety—specifically biodegradation and cytotoxicity—remains a critical challenge [221]. In hydrogels incorporating conductive materials, potential leakage or leaching of constituent polymers can induce severe cytotoxicity, which currently hinders their practical and widespread clinical applications in tissue engineering [222]. Moreover, the use of these platforms is further limited by regulatory uncertainties regarding doping agents and their concentrations, alongside persistent concerns about long-term functional stability [223]. To address these bottlenecks, intensive research is required to establish standardized evaluation metrics and enhance the biocompatibility of conductive hydrogels through stimuli-responsive properties or precise chemical modifications [224,225].

In addition to these material-level limitations, advancing conductive hydrogels for tissue engineering applications requires precise spatialization of structural features tailored to complex, patient-specific wounds, as well as closed-loop responsiveness. Therefore, fabricating wearable patches with controlled configurations and optimized properties by forming 3D complex architectures represents an essential milestone to ensure superior sensing performance [226]. Utilizing 3D printing technologies with conductive hydrogel precursor inks enables the high-resolution manufacturing of custom patterns and delicate hydrogel structures, thereby facilitating seamless deposition onto various substrates [227]. Furthermore, 3D printed conductive hydrogels offer collateral advantages, such as reduced manufacturing costs and streamlined operational workflows. This approach stands as a highly attractive technology capable of significantly maximizing piezoresistive, capacitive, and self-powered efficiencies depending on the specific geometry of the fabricated architecture [228].

## 5. Advanced Technologies for Tissue Engineering

### 5.1. 3D Printing Technologies in Biomedical

3D printing is an additive manufacturing technology that creates complex geometric shapes by depositing materials layer by layer from 3D digital models, and its application in tissue engineering is actively being researched [229,230]. Specifically, when 3D bioprinting is applied to regenerative medicine, it offers significant advantages by enabling the fabrication of scaffolds that mimic complex tissue structures and provide customized responses to specific clinical scenarios [231]. Unlike conventional manufacturing, 3D bioprinting requires a profound understanding of polymer ink processing and post-printing hydrogel curing, as it uses soft, biomaterial-based solvents as inks [232,233]. Furthermore, this technology is highly sophisticated and demanding; the ink must maintain high printing accuracy, structural stability, and biocompatibility during the extrusion process while achieving the target mechanical strength and elasticity [234,235]. Ultimately, the goal is to create growth-promoting structures that enable cell migration and proliferation, thereby enabling the fabrication of functional biomimetic composite tissues and optimal scaffolds within an ideal microenvironment [236].

Extrusion-based 3D printing is the most common method. It involves the layer-by-layer deposition of materials through a nozzle or needle, guided by 3D model data, with consistent movement along the x, y, and z axes. During this process, the outcome is influenced by factors such as extrusion pressure, speed, nozzle diameter, and temperature [237]. Extrusion-based printing is widely used due to its simplicity, versatility, and user-friendliness, enabling rapid, controllable printing and immediate parameter adjustment based on real-time feedback [238]. In this context, the most critical factor for the polymer inks used in this method is viscosity. If the viscosity is too low, the fluid may spray inconsistently or fail to maintain its shape; conversely, if the viscosity is too high, it may clog the nozzle, leading to process failure [239]. Therefore, the viscosity of polymer inks must be carefully prepared by considering multiple parameters (including polymer concentration, molecular weight, solubility, shear rate, and temperature) before the extrusion process [240]. An indispensable and critical characteristic of polymer inks utilized in extrusion-based 3D printing is their rheological behavior [241]. Investigating these rheological properties enables precise screening of polymer inks with varying viscosities to evaluate their suitability for high-fidelity printing, thereby enabling comprehensive quantification of the extrusion-based 3D printing process [242]. Unlike conventional solids, hydrogels display a distinct shear-thinning behavior. In this rheological phenomenon, the material exhibits solution-like fluidity when subjected to shear stress but rapidly recovers to a stable gel state upon removal of the stress. During the 3D printing process, this mechanism ensures that the polymer ink extruded through a micro-nozzle or fine needle immediately regains its structural integrity and mechanical stability, enabling continuous layer-by-layer deposition with high fidelity [243]. In one study, researchers developed a 3D printed hydrogel using GelMA and alginate—biopolymers suitable for extrusion—enhanced with tri-calcium phosphate (β-TCP) to stimulate osteogenic adhesion and differentiation. The resulting hydrogel exhibited non-Newtonian viscous behavior, with viscosity decreasing as shear rate increased, particularly at higher β-TCP contents (0%, 5%, 10%). Additionally, the material showed linear viscoelastic behavior, confirming excellent printability through the nozzle across all experimental groups [244]. Notably, while the pore diameter of the 3D printed hydrogels decreased as the β-TCP content increased, significant cell attachment and proliferation were observed on the hydrogel surfaces.

**Figure 5:**
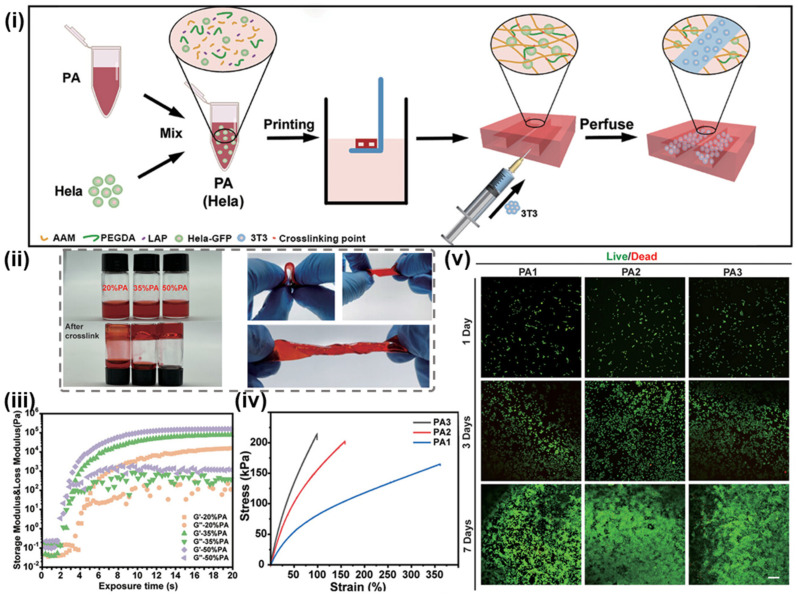
3D printing hydrogels [262]. (**i**) Schematic of the fabrication of cell−laden hydrogel structures using DLP. (**ii**) Comparison of the degree of photocuring and flexibility under identical UV irradiation according to PEGDA−AAm concentration. (**iii**) Rheological properties and (**iv**) mechanical property graphs according to PEGDA−AAm concentration. (**v**) Comparison of cell proliferation rates according to PEGDA−AAm concentration.

Digital Light Processing (DLP)-based 3D printing is a high-resolution technology that fabricates objects layer by layer through light-induced curing [245]. While conventional photosensitive resins used in DLP have limitations regarding biocompatibility and cell deposition, advanced bio-resins are being developed by grafting methacrylate (MA) groups onto biocompatible polymers or adding photoinitiators (e.g., lithium phenyl-2,4,6-trimethylbenzoylphosphinate (LAP)) to hydrogel solvents [246]. DLP printing enables rapid, uniform layer filling, enabling the fabrication of dense 3D structures; however, the build volume is often limited to relatively small sizes due to the restricted exposure area [247]. In one study, a resin containing NIH-3T3 cells was prepared using a PEGDA-acrylamide bio-ink and LAP as a photoinitiator. This facilitated the development of a DLP bioprinting technique that produced structures with high stability and superior elasticity (Figure 5(i)). It was observed that higher concentrations of PEGDA-AAm led to faster curing rates and more flexible behavior post-curing (Figure 5(ii)). Furthermore, rheological analysis showed that the storage and loss moduli improved with higher PEGDA-AAm concentrations; the elastic modulus increased from 45 kPa to 210 kPa, while the strain tended to decrease from 350% to 90% (Figure 5(iii,iv)). Within this PEGDA-AAm environment, NIH-3T3 cells exhibited high cell viability (over 90%) and a daily proliferation rate exceeding 100% as the concentration increased (Figure 5(v)).

Stereolithography (SLA)-based 3D printing is a rapid prototyping process that, like DLP, uses photopolymerization to build complex structures layer by layer [248]. The SLA process typically employs a UV laser (e.g., 355 nm wavelength) to induce radical or cationic photopolymerization, curing the material by tracing precise cross-sections along point-patterned boundaries [249,250]. By controlling parameters such as UV laser power, scanning speed, exposure time, spot size, and wavelength, high accuracy and consistency can be achieved [251,252]. Despite its relatively slow printing speed, SLA produces highly precise outputs and maintains superior cell viability because it exerts the lowest shear stress on encapsulated cells. Consequently, it is considered one of the most promising methods for cell-laden bio-inks [253,254]. In one study, a bio-ink was fabricated using a GelMA and PEGDA base, reinforced with biocompatible nanoparticles (titanium dioxide nanorods (Ti) and kaolinite nanoclay (KLT)) known to improve mechanical strength and inactivate viruses, bacteria, and fungi [255]. Samples printed via SLA using this bio-ink exhibited Young’s moduli ranging from 7.8 kPa to 8.3 kPa and strains of 50% to 46% depending on the nanoparticle addition; remarkably, these ranges align with the mechanical properties of natural human tissues. Moreover, the samples maintained a cell growth rate of over 95% for 1 week, demonstrating the potential to develop implants using low-viscosity biomaterials. In the case of photocurable-based 3D bioprinting, the cross-linked polymeric network is formed through photoinitiator-mediated free-radical chain-growth polymerization [256,257]. However, these highly reactive free radicals can readily interact with crucial cellular components, such as nucleic acids and intracellular proteins, potentially causing significant cytotoxicity. Furthermore, strict attention must be paid to the biosafety concerns associated with UV light exposure during the photocurable printing process [258]. Because the resulting biocompatibility profiles often vary substantially depending on the specific type and concentration of photoinitiators, as well as the target cell types, a customized design strategy tailored to the precise requirements of each biological application is highly required [259,260,261].

### 5.2. Integration of Smart Monitoring Capabilities

**Figure 6:**
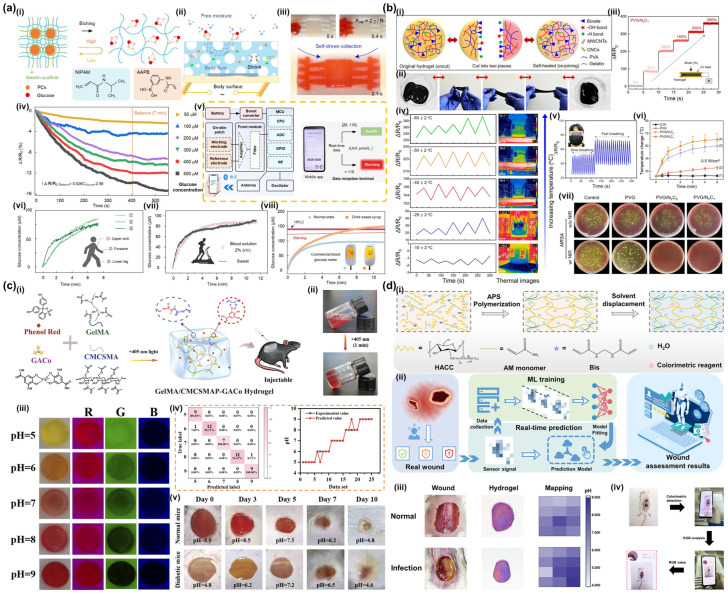
Multimodal and stimuli-responsive hydrogel sensors for monitoring. (**a**) Hydrogel sensor for monitoring glucose in sweat [280]. (**i**) Schematic of the reversible deformation mechanism via boronate ester formation between glucose and AAPB. (**ii**) Schematic of the mechanism converting deformation signals into electrical signals via hygroscopic expansion. (**iii**) Photographs of the spontaneous absorption and behavior of the actual hydrogel. (**iv**) Resistance−time curves of the hydrogel according to glucose concentration. (**v**) Schematic of signal acquisition, wireless transmission, and alarm functions. (**vi**) Signal changes according to the attachment site. (**vii**) Detection results in sweat and blood solutions. (**viii**) Detection curves of the hydrogel before and after the consumption of sweet syrup. (**b**) Multi-functional hydrogel patch for sensing human motion, temperature, and humidity [281]. (**i**) Schematic of hydrogel composition and self-healing mechanism. (**ii**) Photographs showing “slime−like” behavior during the reformation process after deformation. (**iii**) Pressure, (**iv**) temperature, (**v**) respiration−dependent resistance−time curves of the hydrogel. (**vi**) NIR−responsive photothermal reaction curves according to the nanomaterials added to the hydrogel. (**vii**) Antibacterial analysis photographs of the hydrogel against MRSA with and without NIR phototreatment. (**c**) pH−monitoring hydrogel utilizing ML algorithms [282]. (**i**) Schematic of the synthesis of the pH−monitoring hydrogel. (**ii**) Macroscopic observation of hydrogel formation. (**iii**) RGB comparison photographs of the hydrogel according to pH. (**iv**) Confusion matrix of KNN prediction for pH and a comparative analysis between predicted and experimental pH values. (**v**) Real−time pH−monitoring photographs of normal and diabetic wounds over time. (**d**) Wound site monitoring hydrogel utilizing ML algorithms [24]. (**i**) Schematic of the synthesis of the wound monitoring hydrogel. (**ii**) Schematic of the personalized wound management process, from sensor signal collection to ML training and real−time prediction. (**iii**) Wound classification and pH sensing map. (**iv**) In vivo wound healing experimental process under intelligent wound monitoring.

The integration of biosensors into healthcare systems is crucial not only for achieving high sensing sensitivity but also for enabling effective diagnosis and monitoring for timely clinical intervention [263]. In particular, developing systems capable of continuous monitoring—such as for chronic wounds—requires in-depth research to minimize immune responses through seamless integration with physical tissues and to enhance the reliability of signal detection in response to external stimuli (e.g., temperature, light, magnetism) or internal conditions (e.g., ROS, glucose, pH, and enzymatic activity) [264,265]. To ensure compatibility with attached tissues, these devices must possess high flexibility and stretchability. In this regard, ergonomic patches can be fabricated by selecting highly elastic materials or by employing structural designs that balance ductility and mechanical properties, such as serpentine or wavy configurations, to achieve synchronization with the underlying tissue [266,267]. The most critical aspect of a hydrogel sensor is the immediate transduction of biological signals into either optical (colorimetric and fluorescent) or electrical signals [268]. Optical methods capture visual data by detecting reactions in responsive substances (such as pH-sensitive dyes or fluorophores) triggered by changes in environmental factors. This approach allows for intuitive visual identification by the naked eye or, to a more advanced degree, systematic diagnosis through spectrophotometry or imaging processing [269]. In this process, for successful application in tissue engineering, it is essential to evaluate the toxicity and biocompatibility of the responsive materials; furthermore, designers should consider structural color technologies that change hue in response to the contraction and relaxation occurring at the attachment site, beyond simple fluorescence or chemical color changes [270]. Alternatively, electrical sensing methods analyze electrophysiological and mechanical signals with high resolution and high throughput while mitigating impedance mismatches between the sensor and the tissue [271]. These systems can monitor biochemical markers (such as glucose, lactate, or ROS) via electrochemical oxidation-reduction (redox) reactions at the hydrogel-electrode interface, as well as physical parameters, such as structural deformations of the hydrogel matrix induced by external factors (temperature, pressure, and humidity) [272]. In one study, the reaction between glucose and 3-(acrylamido)phenylboronic acid (AAPB) was used to fabricate a wearable hydrogel patch for non-invasive blood glucose monitoring (Figure 6a(i)). Inspired by the moisture absorption mechanism of cactus spines, this biomimetic structure facilitates the diffusion of sweat into the gel; consequently, glucose in the sweat reacts with AAPB, inducing pore deformation due to volume changes, which is then converted into a change in the impedance ratio for simplified signal processing (Figure 6a(ii,iii)). It was confirmed that higher glucose concentrations led to faster moisture absorption and a corresponding increase in relative resistance changes through swelling (Figure 6a(iv)). Additionally, the study developed a wearable device that wirelessly transmits the acquired signals in a low-power data format, allowing for convenient glucose monitoring (Figure 6a(v)). Since this system detects glucose in sweat, it showed consistent measurement patterns across various attachment sites on the arm (Figure 6a(vi)); notably, comparative analysis confirmed a highly consistent trend when sweat and blood were diluted to 2% (*v*/*v*) (Figure 6a(vii)). Finally, the hydrogel patch successfully identified the difference in glucose levels between a fasting state and after consuming sweet syrup, showing results consistent with commercial devices and high-performance liquid chromatography (HPLC) analysis (Figure 6a(viii)). In another study, researchers developed a hydrogel patch with self-healing properties and high stretchability by utilizing the reaction between PVA and borax, inspired by “slime” (Figure 6a(i,ii)). This hydrogel demonstrated multi-functional sensing capabilities by identifying differences in resistance change rates in response to factors such as pressure, temperature, and respiration (Figure 6a(iii–v)). Furthermore, the photothermal properties of the incorporated CNTs were successfully expressed within the hydrogel matrix, showing high antibacterial activity when applied to methicillin-resistant *Staphylococcus aureus* (MRSA) (Figure 6a(vi,vii)).

While the scope of hydrogel-based biosensing is expanding, AI technology has been integrated to enhance monitoring intelligence and functionality, addressing limitations such as low screening efficiency and insufficient structural/characteristic analysis [273]. Hydrogel patches have evolved to measure vital signs, including electroencephalogram (EEG), EMG, and electrocardiogram (ECG); thus, systems for diagnosis and therapy are being established, aiming to advance high-precision feedback through meaningful data analysis [274]. AI or machine learning (ML) techniques can learn from vast datasets and perform predictive modeling, thereby enabling more efficient diagnosis. More importantly, these tools enable the fabrication of highly efficient, customized biosensor hydrogels by analyzing complex interactions governed by the matrix composition (material combinations and crosslinking conditions) [275]. However, because these smart AI-powered wearable patches continuously monitor and transmit sensitive physiological data of patients, establishing stringent data security protocols is imperative [276,277,278]. Furthermore, to ensure accurate diagnosis and timely clinical interventions, consistent patient data must be acquired, and proper maintenance and strict user adherence are required to proactively prevent data gaps or inconsistencies [279].

In one study, specifically, an improved management strategy for accurate pH evaluation was proposed by applying an ML algorithm to a pH-monitoring hydrogel. To impart pH-responsiveness to a GelMA/CMC (CMCSMA) matrix, GA and cobalt ions were used to form polyphenol-metal coordination bonds, resulting in a material that dissociates in response to acidity (Figure 6c(i)). Stable crosslinking was achieved under blue light (405 nm) (Figure 6c(ii)). The resulting hydrogel exhibited distinct color changes within the pH 5–9 range. By training a K-nearest neighbor (KNN) model on RGB signal values, the researchers achieved a 96% prediction accuracy (Figure 6c(iii,iv)). Based on these results, practical in vivo experiments demonstrated that the monitoring model could overcome the limitations of visual color perception, which often struggles to distinguish infection-related changes (Figure 6c(v)). Another study, a strategy integrating precision therapy, real-time monitoring, and personalized care was developed using a hydrogel composed of PAM and chitosan quaternary ammonium salt (HACC-PAM) combined with a convolutional neural network (CNN) model (Figure 6d(i,ii)). Upon recognizing the hydrogel attached to a wound, the system identifies the wound area and performs pH mapping to assess infection and healing progress (Figure 6d(iii)). As a result, the process of wound-healing recognition was simplified by analyzing RGB values from photos of the infected site to obtain immediate clinical results (Figure 6d(iv)).

### 5.3. Strategies for the Commercialization of Hydrogels

Hydrogels, which are widely investigated as highly hydrated polymeric matrices, present critical challenges that must be overcome to guarantee their stability and therapeutic efficacy during practical commercialization [283]. To successfully commercialize hydrogels for tissue engineering applications, several prerequisites must be rigorously evaluated, including whether processing methods alter their physicochemical properties, whether toxic components are introduced during application, and whether host immune rejection occurs [283,284,285]. Specifically, inflammatory responses induced by hydrogels can significantly impair the survival and behavior of encapsulated or transplanted cells, and vice versa, thereby imposing substantial constraints on the selection of materials [286]. Although sterilization is indispensable for hydrogels that interface closely with biological tissues, establishing efficient sterilization protocols without compromising the hydrogels’ inherent properties remains highly challenging [287].

The primary modalities of conventional sterilization include aseptic processing and terminal sterilization. Aseptic processing involves fabricating hydrogels in a sterile environment by ensuring that all raw materials and manufacturing equipment are pre-sterilized [288]. However, this approach requires extreme regulatory controls, faces significant environmental maintenance difficulties, and incurs high operational costs; furthermore, even hydrogels fabricated under such conditions cannot guarantee absolute biological safety, leading to the preferential adoption of terminal sterilization. Terminal sterilization refers to sterilizing the final product within its ultimate packaging, thereby substantially reducing production costs and enhancing sterility assurance [289]. Nevertheless, because exposure to physical or chemical factors during sterilization can alter the mechanical properties of biomaterial-based hydrogels or introduce toxic residues, rigorous evaluations of the risks of degradation and impurity generation are mandatory [290].

Long-term storage stability poses another formidable challenge for hydrogels, which are highly susceptible to structural damage from external factors, such as mechanical forces and temperature fluctuations [291,292]. Although their inherently poor mechanical performance can be improved by increasing crosslinking density to enhance structural rigidity or by using reversible crosslinking mechanisms to fabricate self-healing hydrogels, a critical parameter that requires careful oversight during storage is the high water content within the hydrogel network [293]. In tissue engineering, particularly for drug-loading systems, refrigeration is frequently required for preservation, which necessitates robust anti-freezing properties; moreover, practical in vivo applications must also account for variables such as excessive swelling resulting from prolonged physiological contact [294,295].

For layer-by-layer fabrication processes such as 3D bioprinting, maintaining cross-sectional quality and consistency, and achieving high reproducibility between printed layers is imperative [296,297]. Ensuring these factors are achieved is essential for the efficient and uniform production of hydrogels for reliable clinical applications [298]. Furthermore, to utilize hydrogels as reliable biosensors, reproducing batch-to-batch variation represents another critical bottleneck for successful commercialization [299]. Natural polymers have consistently faced criticism for compromised reproducibility and stability that depend on the raw material source or processing techniques; therefore, these limitations must be thoroughly validated and addressed to establish a concrete rationale for their clinical translation in tissue engineering [300,301].

## 6. Conclusions and Future Perspectives

The paradigm of wound care is undergoing a transformative shift from simple physical barriers to active, intelligent therapeutic systems. As discussed throughout this review, multifunctional hydrogel scaffolds have demonstrated exceptional potential in addressing the complexities of the wound microenvironment by synergistically combining hemostatic performance, antimicrobial activity, and electrical conductivity. However, despite these significant advancements, several critical challenges remain to facilitate widespread clinical adoption. First, the long-term biocompatibility and biodegradability of persistent conductive fillers, such as carbon nanotubes or certain metal nanoparticles, require more rigorous longitudinal studies to determine their metabolic fate and potential systemic toxicity. Furthermore, a complex engineering hurdle remains in striking a perfect balance between the high mechanical stretchability required for wearable sensing patches and the structural stiffness necessary for tissue scaffolding and support. In terms of electronic integration, the current reliance on high-power computing for signal processing and ML models must transition toward low-power, edge-computing solutions that can be integrated directly into wearable hardware for real-time applications. Lastly, as these hydrogel sensors begin to transmit sensitive physiological data, establishing secure communication protocols and navigating the complex regulatory pathways for “software-as-a-medical-device” (SaMD) will be essential for ensuring patient safety and data privacy. Ultimately, the next frontier in medical hydrogel research lies in the development of closed-loop “Sense-and-Treat” systems that autonomously trigger therapeutic interventions in response to real-time microenvironmental changes, thereby redefining the standards of personalized regenerative medicine.

To achieve the successful commercialization of a tissue-engineering hydrogel, a roadmap must be established. The first goals must focus on shifting from complex, expensive multi-step formulations to scalable, standardized synthesis protocols that resolve batch-to-batch structural variations. The second goal must secure automated multimodal sensor calibration, along with robust cybersecurity frameworks for edge computing devices. Looking toward 2030, the ultimate milestone is successfully navigating human clinical trials and securing FDA/CE approvals for automated ‘Sense-and-Treat’ platforms. Currently, the single biggest bottleneck hindering this translational trajectory is the vast biological discrepancy between standard in vivo small-animal models and the actual human chronic wound microenvironment. Standard rodent excisional wounds heal primarily via rapid skin contraction, whereas human chronic wounds are stalled in prolonged inflammation and heal through complex granulation and re-epithelialization. Overcoming this model discrepancy and proving the long-term systemic biosafety of chronic nanomaterial exposure remain the paramount prerequisites for the next decade of advanced wound care.

## Data Availability

No new data were created or analyzed in this study. Data sharing is not applicable to this article.
